# Bacterial vaginosis and cervical human papillomavirus infection in young and adult women: a systematic review and meta-analysis

**DOI:** 10.11606/s1518-8787.2022056004412

**Published:** 2022-11-18

**Authors:** Bruno César Teodoro Martins, Rafael Alves Guimarães, Rosane Ribeiro Figueiredo Alves, Vera Aparecida Saddi

**Affiliations:** I Universidade Federal de Goiás Faculdade de Medicina Programa de Pós-Graduação em Ciências da Saúde Goiânia GO Brasil Universidade Federal de Goiás. Faculdade de Medicina. Programa de Pós-Graduação em Ciências da Saúde. Goiânia, GO, Brasil; II Universidade Federal de Goiás Faculdade de Enfermagem Programa de Pós-Graduação em Enfermagem Goiânia GO Brasil Universidade Federal de Goiás. Faculdade de Enfermagem. Programa de Pós-Graduação em Enfermagem. Goiânia, GO, Brasil; III Pontifícia Universidade Católica de Goiás Programa de Pós-Graduação em Ciências Ambientais e da Saúde Goiânia GO Brasil Pontifícia Universidade Católica de Goiás. Programa de Pós-Graduação em Ciências Ambientais e da Saúde. Goiânia, GO, Brasil

**Keywords:** Bacterial Vaginosis, Epidemiology, Papillomavirus Infections, Risk factors, Revision

## Abstract

**OBJECTIVE:**

To investigate the association between bacterial vaginosis and cervical human papillomavirus (HPV) infection in young and adult women.

**METHODS:**

This systematic review and meta-analysis was based on the Prisma methodological guidelines. PubMed and Web of Science were searched using the following descriptors: “bacterial vaginosis and HPV”, in June 2019. Articles published from 2012 to 2019 were included. Inclusion criteria were original studies that investigated the association between bacterial vaginosis and cervical HPV infection; articles published in English, Spanish or Portuguese; studies conducted in young and adult, non-pregnant, non-HIV-infected women; studies that used the Nugent criteria for the diagnosis of bacterial vaginosis and studies in which the detection of HPV used the polymerase chain reaction technique. Assembled data, odds ratio (OR) and respective 95% confidence intervals (95%CI) were estimated for the association between bacterial vaginosis and cervical HPV infection using random-effects models. A bilateral value of p < 0.05 was considered statistically significant.

**RESULT:**

Six studies were selected for analysis and demonstrated association between bacterial vaginosis and cervical HPV infection (OR = 2.68; 95%CI: 1.64–4.40; p < 0.001).

**CONCLUSION:**

Bacterial vaginosis was considered a risk factor for cervical HPV infection, since women with bacterial vaginosis were more likely to be infected with HPV.

## INTRODUCTION

Bacterial vaginosis has been reported as a cofactor for the HPV infection^1^, being the most common cause of vaginal discharge and affecting about 30% of women worldwide^4,5^. The bacterial infection results from a change in the vaginal microbiota with a decrease in *Lactobacillus spp.*, commonly present in the vaginal environment, with polymicrobial etiology and predominance of anaerobic microorganisms^6,7^.

HPV infection and bacterial vaginosis have common risk factors, including: early onset of sexual activity, history of multiple partners, history of other sexually transmitted infection (STI), and inconsistent condom use^4,8,9^, leading to the hypothesis that both are associated^1^.

Studies have shown a greater diversity of vaginal bacteria in HPV positive women than in HPV negative women^10,11^. The vaginal microbiota plays an important role in women’s reproductive health and serves as the first line of defense against STI^12^. It also seems to have a crucial role in the prevention of HPV infection, since it accelerates the clearance of the virus, so an imbalance of vaginal microbiota could be a synergistic factor for the development of HPV related diseases^12^.

Three meta-analyzes investigated the association between bacterial vaginosis and HPV infection in recent years^1^. Although the results obtained in those studies are important, they included heterogeneous populations and diverse methods for both HPV detection and bacterial vaginosis diagnostic, making it difficult to compare and to interpret the results. In the present systematic review and meta-analysis, we only included declared HIV-negative, young and adult, non-pregnant women. However, all the selected studies used PCR as the chosen method for HPV DNA detection and the Nugent criteria for the diagnosis of bacterial vaginosis, allowing a lower risk of bias in the interpretation of results. Therefore, the purpose of this systematic review and meta-analysis was to investigate the association between bacterial vaginosis and cervical HPV infection in young and adult women.

## METHODS

This systematic review and meta-analysis used the guidelines of the Preferred Reporting Items for Systematic Reviews and Meta-Analyzes: The Prisma Statement (Prisma)^15^ as foundation. Established using an acronym for patient, intervention, comparison, and “outcome” - PICO^16^- the guiding question of the study was: is there an association between bacterial vaginosis and cervical HPV infection in young and adult women? “Patients” were young and adult women with the presence or absence of bacterial vaginosis, and “outcome” indicates the presence or absence of cervical HPV infection.

The databases National Library of Medicine (PubMed/Medline) and Science Citation Index Expanded (Web of Science) were searched using descriptors included in the Medical Subject Headings (MeSH terms): “bacterial vaginosis and HPV”. The search for the studies was carried out in June 2019.

Inclusion criteria: original studies that investigated the association between bacterial vaginosis and cervical HPV infection, articles published in English, Spanish or Portuguese, studies conducted in young and adult women, over 15 years old, non-pregnant, declared HIV-negative, studies that used the Nugent criteria^17^ for the diagnosis of bacterial vaginosis, and studies in which HPV detection has been carried out using the polymerase chain reaction (PCR) technique. Exclusion criteria: studies of systematic reviews, meta-analyzes and case reports. The articles were included regardless of the publication period. All studies were read and analyzed by two independent researchers (BCTM, VAS). Pregnant and HIV-positive women were excluded from this study because these conditions can function as cofactors for bacterial vaginosis and HPV infection, respectively. We only included young and adult women in order to avoid further confounding factors. The diagnostic methods for bacterial vaginosis and HPV established in this study are considered the gold standard for detecting these conditions^17^.

This systematic review was registered in the International Prospective Register of Systematic Reviews (Prospero) under protocol number: CRD42020140790.

Initially, the titles and abstracts were read from each database. Then, the studies pre-selected in the previous step were read in full. From the selected studies, the following information was extracted: authors and year of publication, number of participants in the research, type of study, country of study, mean age, method of diagnosis of bacterial vaginosis and HPV, positivity for bacterial vaginosis and HPV infection in each group.

In order to identify potential risks of bias, studies were assessed based on criteria adopted by Brusselaers et al.^2^ (2019), presented in Chart 1. Two researchers performed the search and reading of the articles and the data collection instrument was completed independently (Chart 1). We subsequently compared the data in order to minimize possible risks of measurement bias (error in interpretation of results and design). After the application of the classification and assessment tool for the quality of the articles (Chart 2), a score of up to 8 points was assigned to each study. No divergence regarding the evaluation of publications was reported.

**Chart 1:** Tool for classification and evaluation of the quality of included articles.

Criteria	Score
The initial study had a cross-sectional or cohort;	1
The study sample was representative for the specified population, reflecting the community or based population - sample calculation;	1
The evaluation of bacterial vaginosis was performed by microscopy based on Nugent’s criteria;	1.5
HPV detection was performed by PCR;	1.5
The study was properly developed in relation to the association between bacterial vaginosis and HPV detection;	1
HPV positive cases were classified as high and low risk or in specific genotypes;	1
The potential confounding factors (smoking, parity, hormonal contraception, concomitant STI, age, number of partners), were properly assessed and accounted for the analysis.	1

HPV: human papillomavirus; PCR: polymerase chain reaction; STI: sexually transmitted infection.

Source: Adapted from Brusselaers et al.^2^ (2019).

**Chart 2:** Degree of evidence of the studies according to the classification and evaluation tool.

A total of 8 points can be assigned to each study, categorized as:
1–3: high risk of bias (poor quality evidence)
4–5: moderate risk of bias (moderate quality evidence)
6–8: low risk of bias (high quality evidence)

Source: Adapted from Brusselaers et al.^2^ (2019).

This meta-analysis with the objective of verifying the association between bacterial vaginosis and HPV infection was conducted in the Stata program, version 15.0. Initially, the study estimated the prevalence of HPV in both groups (with and without bacterial vaginosis). Assuming that I^2^ values of 25, 50, and ≥ 75% represent low, medium and high heterogeneity, respectively, we used this statistic to verify the heterogeneity between the studies. For combined estimation of study data, odds ratio (OR) and respective 95%CI were calculated for the association between bacterial vaginosis and cervical HPV infection, using random effects models and the results were presented in forest plot. A bilateral p < 0.05 value was considered statistically significant.

## RESULTS

**Figure 1:**
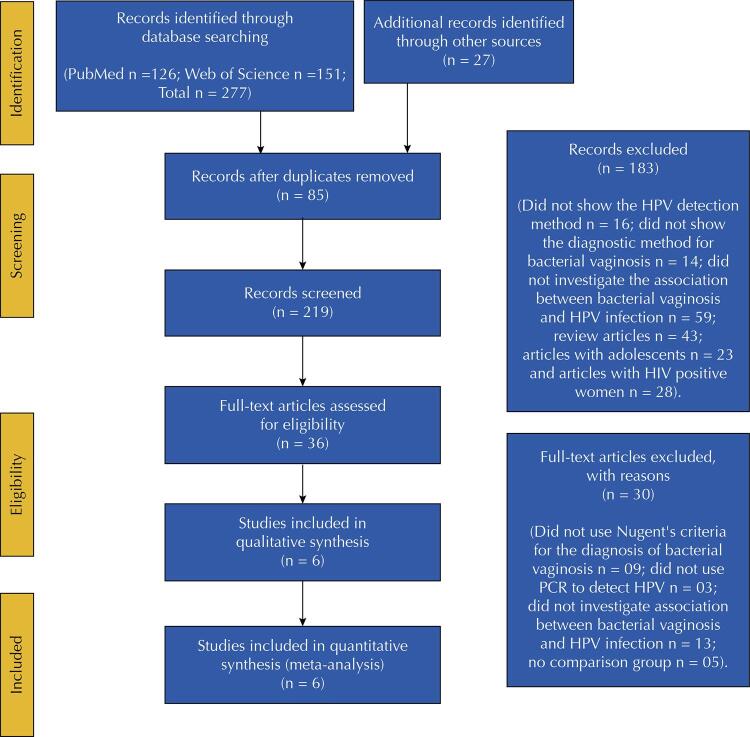
Flowchart of the studies selection for the systematic review and metanalysis (Prisma 2020 flow diagram)

Six articles published in English from 2012 to 2019 were included in the study, as described in Figure 1. Table 1 summarizes the results. This meta-analysis assessed a total of 7.119 women (1.131 cases and 5.988 controls). In each study the number of them ranged from 181 to 3.502 and their average age ranged from 16 to 38.5 years. Only one study had a cohort design^20^, and the others were cross-sectional studies. From the included studies, only two evaluated the association between bacterial vaginosis and CIN^21,22^. Asia (33.3%) and America (33.3%) conducted most of them with two studies in each continent, then one in Europe (16.7%), and one in South Africa (16.7%), as shown in Table 1.

Page MJ, McKenzie JE, Bossuyt PM, Boutron I, Hoffmann TC, Mulrow CD, et al. The PRISMA 2020 statement: An updated guideline for reporting systematic reviews. BMJ 2021;372:n71. doi: 10.1136/bmj.n71.

HPV: human papillomavirus; PCR: polymerase chain reaction.

**Table 1:** Characteristics of the six studies included in the systematic review.

Authors and year of publication	Number of participants	Study design	Country	Mean age	HPV detection method	BV detection method	BV (+)		BV (-)	
	
							HPV +	HPV -	HPV +	HPV -
Oakeshott et al.^20^ (2012)	2075	Cohort study	England	21 years	HPV DNA (PCR)	Nugent criteria	133 (25.8%)	382 (74.2%)	258 (16.5%)	1302 (83.5%)
Caixeta et al.^21^ (2015)	251	Cross-sectional study	Brazil	19.4 years	HPV DNA (PCR)	Nugent criteria	59 (57.23%)	44 (42.7%)	52 (35.1%)	96 (64.9%)
Lu et al.^22^ (2015)	3502	Cross-sectional study	China	38 years	HPV DNA (PCR)	Nugent criteria	270 (83.3%)	54 (16.7%)	1468 (46.2%)	1710 (53.8%)
Mongelos et al.^23^ (2015)	181	Cross-sectional study	Paraguay	30 years	HPV DNA (PCR)	Nugent criteria	18 (31.5%)	39 (68.5%)	24 (19.3%)	100 (80.7%)
Mbulawa et al.^24^ (2018)	284	Cross-sectional study	Africa do Sul	16 - 22 years	HPV DNA (PCR)	Nugent criteria	31 (77.5%)	09 (22.5%)	157 (64.3%)	87 (35.7%)
Panpan et al.^25^ (2019)	826	Cross-sectional study	China	38.5 years	HPV DNA (PCR)	Nugent criteria	52 (56.5%)	40 (43.5%)	202 (27.5%)	532 (72.5%)

After applying the classification tool and evaluating the quality of the articles, we classified all studies as low risk of bias, with scores between 6 and 8, therefore, presenting high quality evidence.

**Figure 2:**
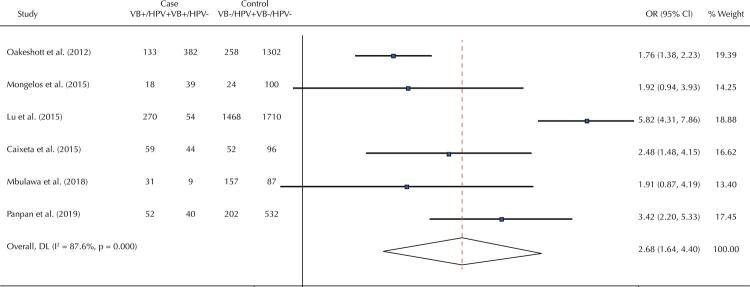
Forest plot of the studies included in the meta-analysis of the association between bacterial vaginosis and HPV infection.

HPV: human papillomavirus; PCR: polymerase chain reaction.

Figure 2 shows the association between bacterial vaginosis and HPV infection through a random effect meta-analysis. The pooled estimate showed a significant association between bacterial vaginosis and cervical HPV infection (OR = 2.68; 95%CI: 1.64–4.40; p < 0.001). Therefore, women with bacterial vaginosis were 2.68 times more likely to have cervical HPV infection when compared with women without bacterial vaginosis. Evidence of heterogeneity was found between studies (I^2^: 87.8%; p < 0.001).

## DISCUSSION

The present systematic review and meta-analysis investigated the relationship between bacterial vaginosis and cervical HPV infection, demonstrating a significant association between these conditions. When compared with women without bacterial vaginosis, the random effect model showed that women with bacterial vaginosis were 2.68 times more likely to have cervical HPV infection. The outstanding of our systematic review and meta-analysis is the inclusion criteria of the selected studies comprising those that investigated only declared HIV-negative, young and adult, non-pregnant women. All the selected studies used PCR as the chosen method for HPV DNA detection and the Nugent criteria for the diagnosis of bacterial vaginosis. Furthermore, this study followed PRISMA recommendations in this review, and obtained Prospero registration and certification.

Performing a sensitive and specific exponential amplification of a fragment of the virus genome^18,19^, the PCR method uses enzymatic synthesis of viral DNA. Nevertheless, the Nugent criterion consists of counting bacterial morphotypes in a smear stained by the Gram method, quantified and classified using a score, mainly based on the presence or absence of *Lactobacillus spp.*^17^.These methods were used as the inclusion criteria because they allow more accurate diagnosis, with greater sensitivity and specificity^17^.

Bacterial vaginosis appears to be associated with an increased risk of cervical HPV infection and the inverse relationship (cervical HPV infection associated with an increased risk of bacterial vaginosis) also appears to be consistent^1^. Three systematic reviews with meta-analyses developed in recent years showed a significant association between bacterial vaginosis and cervical HPV infection, since the chance of women with bacterial vaginosis acquiring a cervical HPV infection in these studies ranged from 1.33 to 2.62^1^. However, these studies are heterogeneous, as they compare different patient profiles with positive results for other STIs, different methods of detection of HPV and diagnosis of bacterial vaginosis^1^, which makes consistent conclusions difficult.

HPV has several evasion mechanisms from the immune system, which can favor changes in the vaginal epithelium, causing imbalance in the local microbiota, degradation and alteration of the cervical mucus and the appearance of bacterial vaginosis^2,26^. The mechanism by which bacterial vaginosis can influence the acquisition of cervical infection by HPV remains unknown, however, there are some hypotheses including the presence of inflammatory cytokines in the vaginal environment due to a decrease in *Lactobacillus spp.,* and the proliferation of microorganisms as a result of changes in the vaginal microbiota^27^. The degradation of mucin as a consequence of the increased production of some enzymes – especially sialidase – is responsible for breaking down cervical mucus components, leading to bullous discharge that covers the vagina and further favors the degradation of the lining layer of the cervical epithelium, which may cause microlesions and changes in epithelial cells^1,30^. Such enzymes can promote virulence through the destruction of the mucosal protective barrier and, therefore, increase the susceptibility to cervical infection by HPV, facilitating the adhesion, invasion and, eventually, incorporation of HPV oncogenes in the genome of cells in the transformation zone cervical^1,30^.

Furthermore, other effects include changes in the cervical mucosal barrier^30^ and release of volatile amines, which also increases levels of oxidative stress^31^. Another hypothesis is that the anaerobic bacterial metabolism observed in bacterial vaginosis produces ammonia and ammonium nitrite in vaginal secretions, both with carcinogenic potential, can cause changes in cervical epithelial cells, such as exfoliation and transformation^3^.

A significant association was described between bacterial vaginosis and cervical HPV infection in the present systematic review and meta-analysis. These findings reinforce the need for healthcare professionals to carefully investigate the presence of bacterial vaginosis in young and adult women in association with cervical HPV infection and related injuries.

The main limitation of this study is the fact that it included only studies published and available in the two consulted databases, which may have caused publication bias, due to the tendency to selectively publish statistically significant results. In addition, the lack of some data in the literature can also lead to a bias in the results.

One of the main complaints of women who visit the gynecologist is the presence of vaginal discharge, however, for many times an accurate diagnosis of this symptom is not achieved, resulting in an increase in periodic consultations. Bacterial vaginosis can appear as a recurrent condition, implying in a careful view of this condition by the medical professional. We believe that diagnosis and effective treatment of women with this condition can contribute to reduce cervical HPV infection. Bacterial vaginosis was considered a risk factor for cervical HPV infection, since women with bacterial vaginosis were more likely to be infected with it.
